# Sport officials’ use of observational learning

**DOI:** 10.3389/fspor.2024.1289455

**Published:** 2024-01-16

**Authors:** David J. Hancock, Amanda M. Rymal

**Affiliations:** ^1^ School of Human Kinetics and Recreation, Memorial University of Newfoundland, St. John’s, NL, Canada; ^2^ Department of Kinesiology, California State University, San Bernardino, San Bernardino, CA, United States

**Keywords:** observation, officials, skill, sport, practice, performance

## Abstract

**Introduction:**

Observational learning is a key tool for improving skilled performances. Sport officials (e.g., referees, umpires, and judges) might glean particular benefits from using observation, as most officials do not engage in traditional practice. Unfortunately, little is known about how observational learning can be of benefit to sport officials. Thus, the purpose of this study was to take an exploratory approach to learn more about sport officials' use of observation.

**Methods:**

Participants included 206 sport officials (170 male, 35 female, 1 not specified) from 17 sports (mainly ice hockey, soccer, lacrosse, and volleyball). Sport officials completed a 50-question online survey regarding their use of observational learning. Survey questions revolved around the reasons for using observation (e.g., to learn about positioning or rule application), along with when and how participants used observation (e.g., before versus after competitions; watching an unskilled versus skilled model).

**Results:**

Participants used observation most frequently to learn knowledge and application of rules, personality and game management, and fitness and positioning/mechanics. Results revealed that participants preferred to use observation after their competitions, while watching other sport officials in-person, and while observing a skilled model who was correctly executing their tasks.

**Discussion:**

In the discussion, we expand on the results, connecting it to previous research in sport officiating or observational learning. Lastly, we offer suggestions for future researchers that should help build our understanding of sport officials' use of observation.

## 1 Introduction

Observational learning is a process by which individuals view either themselves or someone else to gain information regarding the observed behavior (1). The information gleaned can relate to obtaining physical skills, behavior patterns, judgmental standards, cognitive competencies, and generative rules of behavior (2). There are many forms in which one can observe; when observing the self this can be viewed in the form of self-observation (i.e., current skill level) or self-modeling (i.e., eliminating errors) (3). There are many more options for viewing someone else; specifically, one can view a skilled, unskilled, or learning model, as well as a coping or mastery model (4). No matter which technique one chooses, there is a strong body of literature suggesting that observing any demonstration is beneficial for improving overall performance [for a review, see (5)].

The two main perspectives that explain the benefits of observation for skill acquisition are Gibson’s (6) direct perception perspective and Bandura's social cognitive theory. This research adopts the latter perspective however the authors direct readers to Scully and Newell’s (7) work if interested to review. For Bandura's (2) social cognitive theory, it is suggested that we learn through symbolic coding of the observed behavior and then translate those codes into cognitive representations to later guide our behaviors. This view goes on to suggest that an individual learns through observation based on four processes: attention, retention, behavior reproduction, and motivation. Specifically, the learner must actively attend to the information presented from the observed behavior. The information picked up must then be retained in the form of a cognitive representation that is used as a blueprint to guide their future behavior when reproducing that same movement. Motivation is important because the observer must be motivated to attend to the relevant information, remember the information, and practice the skill in order for it to be learned. Lastly, the learner will use their cognitive representation to guide their motor reproduction. Past research has aligned with this perspective and has supported the notion of a cognitive representation influencing subsequent actions, recall and recognition, and response selection [e.g., (8)]. Researchers adopting this perspective have also shown evidence to support that observational learning is beneficial in acquiring not just motor skills, but also enhancing performance, creating strategies, and assisting in mental states (5)—hence we adopted this perspective for the research herein.

Stemming from the social cognitive theory, Cumming et al. (9) developed the Functions of Observational Learning Questionnaire (FOLQ). The survey assesses athletes’ use of observational learning across three main functions: skill (i.e., using observation to improve sport-specific techniques), strategy (i.e., using observation to improve sport-specific tactics), and performance (i.e., using observation to improve mental skills). Researchers have distributed the FOLQ [e.g., (10, 11)] to glean a better understanding of individuals’ motivations to engage in observational learning (i.e., improving skills vs. strategies vs. performance). These studies, however, provide little insight into *how* observation happens. Addressing this, Ste-Marie et al. (12) developed an applied model for the use of observation. Their model provides a framework that practitioners might consider when designing sport observation interventions. This includes contemplating critical observer and task characteristics [e.g., model similarity; (13)], the environment [e.g., where or when observation happens; (14)], the function [e.g., the purpose of observation; (9)], and other relevant contextual considerations [e.g., modality, instructional features, viewing angle, and timing; (15)].

Throughout the research on observational learning in sport, researchers have primarily focused attention on athletes’ use of observation. Ste-Marie and Hancock (16) rightly noted that the benefits of observation extend to coaches and—the focus of this study—sport officials [i.e., referees, umpires, judges, and officials; (17)]. Many sport officials must develop skills, strategies, and mental toughness to perform their tasks. Mascarenhas et al. (18) outlined five characteristics that underpinned sport officials’ performances, though not all apply to each type of sport official. First, *fitness and positioning* refers to the physical ability and knowledge of where to position oneself in the competition in order to meet the demands of their sport and role. Second, *knowledge and application of the law* implies that sport officials must possess an excellent understanding of their sport rules to effectively adjudicate competitions. Third, *personality and game management* are the communication skills required of sport officials that allow them to maintain quality interactions with coaches and athletes, especially during stressful situations. Fourth, *contextual judgment* is the process whereby sport officials understand when to apply (or not apply) certain rules depending on the nature of a competition (e.g., being lenient during a soccer throw-in when one team is down several goals with only a few minutes to play). Fifth, *psychological features* are the skills sport officials must possess to stay calm, focused, and confident during competitions. Critically, these five characteristics are *learned* by sport officials, rather than innate. However, it is unclear how sport officials learn their skills.

In most contexts, a typical learning method is engaging in practice. Unfortunately, many sport officials operate in “practice-poor” environments (19, 20); that is, unlike athletes, there are relatively few opportunities for sport officials to engage in structured or deliberate practice. This is especially true for novice, intermediate, and sub-elite sport officials, whose “practice” environments are typically the competitions they officiate. As such, it is imperative for sport officials and their organizations to consider alternative methods for learning and developing their requisite skills. One such method is engaging in observational learning, which has shown some promising results.

Hancock et al. (21) conducted one of few studies exploring sport officials’ use of observational learning. Therein, the authors adapted the FOLQ for sport officials and compared the results to athletes and coaches. Sport officials used the skill function the most, followed by the strategy and performance functions—a similar pattern to athletes and coaches. Differences emerged across sport roles: coaches employed the skill and strategy functions more than athletes and officials, while officials used the performance function more than coaches. The researchers concluded that differences were likely a result of the unique roles of athletes (performing skills), coaches (teaching skills and game strategies), and officials (managing and adjudicating competitions). In a follow up study, St. Germain et al. (22) examined other ways in which sport officials used observation, discovering that self-presentation (i.e., commanding presence and demonstrating competence) and communication (i.e., effectively interacting with athletes, coaches, and other officials) were important elements developed through observation.

These two studies constitute the extent of the literature on sport officials and observational learning, and each study has several limitations. Hancock et al. (21), for instance, is limited in that it focuses solely on the functions of observation, using a measure designed for athletes and adapted for sport officials. While this was a necessary first step in the field, it remains a limitation of their findings. Similarly, St. Germain et al. (22) explored only 20 sport officials’ responses to one open-ended question. Their work clearly identified two new areas for observation (i.e., communication and self-presentation), but they did not offer a comprehensive examination into all the areas in which observation could be used, nor how observation is employed by sport officials. Simply, these studies do not offer enough evidence from which organizations can create strategies for enhancing sport officials’ use of observation. Calls by previous researchers [e.g., (5)] are, thus, renewed here. Specifically, more attention ought to be directed to understanding the who, when, where, why, and how of sport officials’ observation. There should also be concerted efforts to explore observation among different sport officiating populations (e.g., by gender and competitive level). Such work is a direct response to Ste-Marie et al.'s (5) call for more research outside of athlete populations.

It is clear that observation learning is a tool for sport officials, which is leveraged to learn skills, strategies, mental performance, presence, and communication. Also evident is that the extant literature reveals a notable gap in our understanding of sport officials’ use of observation. Little is known about the specific types of observation in which sport officials engage (e.g., observing positioning vs. communication). Similarly, there is no evidence indicating how frequently sport officials use observation, nor any evidence on who (e.g., experts vs. same skill level), where (e.g., in-person vs. on television), or when (e.g., before vs. after competitions) they engage in observational learning. Clearly, there is a demonstrated need to study observational learning among sport officials. Rather than distributing the FOLQ to learn about the purpose of sport officials’ observation (i.e., the functions of observation), it was deemed imperative to understand how, when, and why sport officials used observation. This approach more closely aligns with Ste-Marie et al.'s (12) applied model of observational learning and takes a more generalized, comprehensive approach to understanding sport officials’ observation. Thus, the purpose of this study was to broadly explore sport officials’ use of observation. The first research question was, when engaging in observational learning, are there certain elements related to officiating performance for which sport officials use observation differently? Second, how do sport officials participate in observational learning (who, when, and where)? Third, does the use of observation for sport officials differ by the officiated sport or competitive level?

## 2 Methods

All procedures described herein were approved by the Research Ethics Board at the lead author’s university.

### 2.1 Participants

Participants were recruited through social media advertising and by emailing officiating organizations from across Canada—specifically those that had websites with email contact information—asking them to distribute the study notice to their active officials. Our sole inclusion criteria were 18 years of age or older and an active sport official. This process yielded a sample of 206 active sport officials (*X*_age_ = 41.05 years, *SD* = 14.77; *X*_experience_ = 16.22 years, *SD* = 10.42). There were 170 (82.52%) male and 35 (16.99%) female participants; one participant did not indicate sex/gender. Participants were asked to indicate the primary sport they officiated, which resulted in 17 identified sports. The six most popular sports were ice hockey (*n* = 61), soccer (*n* = 33), lacrosse (*n* = 31), volleyball (*n* = 25), baseball/softball (*n* = 15), and basketball (*n* = 14). Lastly, the sample ranged from recreational to professional sport officials, with club/varsity (i.e., officiating competitive youth/early adult athletes) most represented (*n* = 94).

### 2.2 Data collection

Participants completed a three-section, 50-question survey administered through Qualtrics. Prior to the main survey, participants responded to basic demographic information questions (results reported above). For the first section, we developed 23 questions to assess what sport officials learn through observation. The questions were specific to sport officiating tasks, derived from previous research on sport officials’ skills [i.e., (18)] five characteristics for sport officiating performance) and observation [i.e., St. Germain et al.'s (22)] discovery of communication and presentation). An example is “As an official, I use observation to learn about staying focused during competitions.” Responses fell on a Likert scale from 1 (strongly disagree) to 9 (strongly agree). In the second section, we created 17 questions to assess how observation occurred. These questions included when observation happened (e.g., “As an official, I use observation before games I am officiating”), who was observed (e.g., “As an official, I use observation by watching officials who are below my skill level”), and in what context the observation took place (e.g., “As an official, I use observation when I am alone”). Again, responses were recorded on a Likert scale from 1 (strongly disagree) to 9 (strongly agree). For the final section, we generated 10 questions soliciting information on how often sport officials use observation (e.g., “In an average month as an official, how many times do you observe other officials on TV?”). Participants responded with the number of times or hours they used a specific type of observation. Given the nature of this exploratory survey, we have included all questions in the Appendix.

### 2.3 Data analysis

The first section of the survey had 23 questions that were grounded in previous literature on the main elements on which sport officials’ performances can be assessed [e.g., (18, 22)]. Those 23 questions fell into six main categories of officiating performance; as such, we created six variables to use in our analysis: (a) knowledge and application of rules (KAR); (b) personality and game management (PGM), (c) fitness, positioning, and mechanics (FPM); (d) contextual judgement (CJ); (e) psychological characteristics (PC); and (f) officiating and supervisor communication (OSC). Though our intention was not to create a validated survey—instead focusing on exploring an under-researched area in the hopes of setting a future research agenda—we calculated Cronbach's alpha scores for each created variable: (a) KAR = .656; (b) PGM = .805; (c) FPM = .517; (d) CJ = .770; (e) PC = .836; and (f) OSC = .837. This means FPM was “unacceptable”, KAR was “acceptable”, CJ was “good” and PGM, PC, and OSC were “better”. Normally, the FPM score would disqualify it from further analysis. The authors deliberated removing it from analysis vs. keeping it as a variable in the study. Ultimately, we elected to report the results. The rationale was that there is virtually no empirical research on sport officials’ use of observation, so any information that is available to researchers should be shared. However, we recommend researchers interpret the FPM results cautiously—specifically, we recommend the results be considered if planning new research studies in the area, but not quoted as a reliable result.

All data were analyzed using SPSS 26.0. In the results, we report descriptive statistics, showcasing the mean scores for the created variables. After, we move into inferential statistics—specifically, paired samples *t*-tests were used to compare mean scores between the created variables. Additionally, we compared mean scores on other collected variables, such as the use of observation before vs. after competitions. Lastly, we utilized two, one-way ANOVAs to ascertain if mean scores on what officials observed (i.e., the six newly created variables) differed based on officials’ (a) sport and (b) competitive level. Where significant results existed, we ran Tukey's HSD post-hoc test to identify group differences.

## 3 Results

Results herein follow the order of the three research questions for the study.

### 3.1 Sport officials’ use of observational learning

The first set of results reflect what types of observation participants engaged in to improve their sport officiating performances. Table 1 outlines mean scores for the six created variables, ranked from highest to lowest on the 9-point Likert scale. To determine which scores were rated significantly higher than others, we performed paired samples *t*-tests on all pairs of the created variables. It would be unwieldly to show the statistics for all significant tests, so note that the significant pairs below had *p*-values <.001 (with the exception of KAR-FPM, which was <.05) and Cohen's *d* ranged from 0.16 to 0.76, though all but KAR-FPM, PC-OSC, and PC-CJ were above 0.30). KAR was significantly higher than FPM, OSC, CJ, and PC. PGM was significantly higher than OSC, CJ, and PC. FPM was significantly higher than OSC, CJ, and PC. Lastly, PC was significantly lower than OSC and CJ.

**Table 1:** Mean scores for categories of observation.

Variable	Mean	SD
Knowledge and application of rules (KAR)	7.29	1.45
Personality and game management (PGM)	7.14	1.29
Fitness, positioning, and mechanics (FPM)	7.07	1.23
Officiating and supervisor communication (OSC)	6.57	1.67
Contextual judgement (CJ)	6.54	1.88
Psychological characteristics (PC)	6.09	1.69

### 3.2 How sport officials use observational learning

The second set of results reflect our efforts to understand the when, who, and where related to sport officials’ observation—once again employing paired samples *t*-tests. Participants indicated that they used observation significantly more after their competitions (*M* = 7.40, *SD* = 1.56) than before [*M* = 6.89, *SD* = 1.99; *t*(197) = 3.77, *p* < .001, *d* = 0.29] or during [*M* = 6.90, *SD* = 2.48; *t*(197) = 2.47, p < .05, *d* = 0.25] competitions. Next, participants rated watching other sport officials in-person (*M* = 7.95, *SD* = 1.45) significantly higher than watching officials on television [*M* = 7.70, *SD* = 1.52; *t*(197) = 2.53, *p* < .05, *d* = 0.17], watching recordings of other officials [*M* = 7.53, *SD* = 1.54; *t*(195) = 3.93, *p* < .001, *d* = 0.28], and watching recordings of themselves [*M* = 7.52, *SD* = 1.86; *t*(197) = 3.25, *p* < .01, *d* = 0.23]. Third, we assessed the skill level of participants’ preferred type of sport official to observe. Therein, we discovered that participants gave significantly higher ratings to observing sport officials above their skill level (*M *= 8.35, *SD* = 1.24) than they did for observing officials at [*M* = 7.63, *SD* = 1.51; *t*(197) = 8.85, *p* < .001, *d* = 0.63] or below their skill level [*M* = 6.61, *SD* = 2.28; *t*(196) = 11.77, *p *< .001, *d* = 0.84]. Similarly, participants preferred observing sport officials who were correctly executing their skills and strategies (*M* = 8.04, *SD* = 1.34) over those who were making errors [*M *= 7.65, *SD* = 1.70; *t*(198) = 4.12, *p* < .001, *d* = 0.29]. Lastly, participants more frequently used observation in their officiating groups (*M* = 7.70, *SD* = 1.44) than when they were alone [*M* = 7.37, *SD* = 1.80; *t*(197) = 2.49, *p* < .05, *d* = 0.18].

### 3.3 Comparing sport officials’ observation by sport and competitive level

The final analysis was a series of one-way ANOVAs aimed at comparing types of observation (i.e., the six created variables) across sports and competitive levels. To compare across sports, we isolated the six with the most participants (i.e., ice hockey, soccer, lacrosse, volleyball, baseball/softball, and basketball. Lacrosse officials (*M *= 7.20, *SD* = 1.48) used observation for CJ significantly more than ice hockey officials [*M* = 5.98, *SD* = 2.04; *F*(5, 170) = 3.19, *p* < .01, *η*^2^ = 0.09]. No other significant differences existed based on sport. Further, types of observation used by sport officials did not differ based on the competitive level they officiated (all *p*-values > .05).

## 4 Discussion

Since the use of observation among sport officials is relatively unexplored, we endeavored to take a global, somewhat descriptive approach to studying it. Research in this area is imperative, as sport officials serve an important role within organized sport yet operate in practice-poor environments (19, 20); thus, understanding methods by which sport officials can develop is critical. Specifically, 206 active sport officials responded to our comprehensive survey that explored types of observation, along with the who, when, where, and how of observation. In the following paragraphs, we unpack the results of each research question.

### 4.1 Sport officials’ use of observational learning

The first research question attempted to provide insights into sport officials’ use of observation, which was guided by the five characteristics for officiating performance (18) and observational research on officials (22). Our results indicated that sport officials engaged in observation the most for KAR, PGM, and FPM. For two reasons, this result should not be particularly surprising. First, previous researchers have identified rule knowledge (23), decision-making (24), positioning (25), and communication (26) as fundamental sport officiating skills, on which, other relevant skills are built (e.g., CJ). It is why the variables constitute three of the four cornerstones for quality sport officiating (18). The second explanation for the result is that KAR, PGM, and FPM are the most observable behaviors to other sport officials. As noted in the term, observational learning requires action to be observable. Certainly there are instances when communication with a supervisor or mental skills (e.g., deep breathing) could be observable to other sport officials, but quite likely, the frequency of observable KAR, PGM, and FPM is higher. The results do not mean that OSC, CJ, and PC are unimportant skills for sport officials, nor do they mean that officials deprioritize those skills. Simply, our finding indicate that observation is not used as frequently to learn those skills.

Sport organizations that wish to offer development opportunities for their officials might be well served to create observation opportunities (e.g., videos) that officials could view, with an emphasis on KAR, PGM, and FPM. To make OSC, CJ, and PC more “observable”, sport organizations could supplement such videos with audio recordings of expert officials—akin to a think-aloud protocol—whereby observers could watch and listen to learn via observation. Another idea is to have experienced officials demonstrate (i.e., live or on video) the skills they use for officiating (e.g., demonstrating the positive effects of deep breathing on heart rate via biofeedback). Such approaches leverage skilled models (27) to enhance observational learning.

The results related to this research question provide a more nuanced understanding of what sport officials observe. Sport scientists, however, have plenty of avenues for future research that would strengthen our knowledge in this area. Based on our results, a potential study could be to examine if learning the six variables herein is facilitated through an observation intervention, which is a frequently used paradigm with athletes [e.g., (10)]. Further, to strengthen the limited knowledge in this field, researchers could consider longitudinal designs to explore changes in observational learning over time, as well as larger and more diverse sample sizes (e.g., across many countries).

### 4.2 How sport officials use observational learning

Through our second research question, we aimed to build a baseline understanding of when/who/where sport officials observe, which Ste-Marie et al. (12) have called for within and outside of athlete populations. Participants indicated they were more likely to use observation after their competitions, with a group of other sport officials, and while watching officials in-person who were above their skill levels and correctly performing their skills and strategies. These results very much resemble a reflective learning environment. One can imagine an ice hockey referee (as an example) performing, then watching part of the next game. The original referee might watch with their officiating team, and as is often the case with ice hockey, the subsequent officiating crew is operating at a higher competitive level [e.g., a mastery/skilled model; (27)]. Essentially, the original referee has an opportunity to learn the craft by observing others. Since sport officials get little in the way of traditional practice (19, 20), adopting this type of observational learning likely offers notable performance benefits. It is worth stating that engaging in observation after a performance runs counter to most observational learning interventions where video is provided prior to or during physical practice (5). Potentially, this is more effective for sport officials as they do not have a true practice environment and they are not executing a pre-defined performance (like you might see from a gymnast). Regardless, it lends credence to idea that researchers ought to continue studies on the timing of observational learning [e.g., (28)].

Certainly, these results on how sport officials engage in observational learning are preliminary, though they offer excellent insights that sport scientists and organizations can leverage. For sport scientists, a key next step is to explore preferred vs. effective observation. In this study, participants indicated a preference for observing after competitions, in-person, and with skilled models. That does not mean, though, that it is the most effective way to use observational learning. Research that creates observation interventions to determine efficacy of learning would be incredibly valuable. For sport organizations, the results herein emphasize the value of creating videos for sport officials to observe. Specifically, videos of skilled sport officials would likely be received well by the officiating community, perhaps leading to positive learning benefits. Furthermore, since participants used observation to learn KAR, PGM, and FPM (i.e., fundamental skills), it is not surprising that a skilled model was favored.

### 4.3 Comparing sport officials’ observation by sport and competitive level

Officials across sports have varying demands. The head soccer referee operates in the middle of the pitch, whereas a gymnastics judge sits on the outside of the competitive environment. Furthermore, the skills required to be a professional basketball referee likely differ greatly to those required to be an effective grassroots basketball referee. Given such differences, our third research question compared the use of observation by sport and competitive level. Surprisingly, there was very little difference in participants’ responses based on sport or competitive level. In fact, only one test showed a significant difference, with lacrosse officials using observation for CJ more than ice hockey officials. Given the similarity in the two sports and the requirements of the officials, this result is quite unexpected. Rather than focus our attention on this one test, we instead put to the forefront the collective result for this research question; that is, the way in which sport officials use observation does not seem to differ based on the officials’ sport or competitive level. This is a significant result, as it implies that any observation intervention found to be effective for officials in one sport is likely to be effective for officials in other sports. To our knowledge, no such interventions exist in the current literature on sport officials, but as the field grows, organizations looking to develop observation learning strategies can leverage existing knowledge in the field to use within their sports.

It should come as no surprise, then, that our recommendation for sport scientists is to conduct intervention studies with sport officials, similar to those that exist with athletes [e.g., (10)]. Then, as such studies become available, sport organizations can use those results to enhance observational learning opportunities for their officials. We also suggest that if sport organizations implement an intervention that is deemed useful and positive for learning, that it be shared among sport organizations for widespread implementation.

## 5 Conclusions

In conclusion, this research provides new insights into why sport officials use observation techniques and how officials employ observation. It is evident that sport officials use observational learning beyond what is measured by the FOLQ. In fact, sport officials appear to use observation for reasons that enhance or influence many of the qualities deemed necessary for officiating (18). Specifically, their use of observation was related to knowledge and application of rules; personality and game management; and fitness, positioning, and mechanics. The remaining qualities (contextual judgement, psychological characteristics, and officiating and supervisor communication) were less used. However, this might be a result of the various sports officials work in, as opposed to lack of effectiveness—not to mention that these qualities were still rated highly by participants. Based on the results, sport officials tend to prefer to observe differently than sport scientists and researchers presume. That is, sport officials prefer to observe other officials at a higher skill level, after they have already officiated a game, and in-person—giving support to the notion of self-control of viewing in the officiating realm. Competitive level, on the other hand did not seem to influence how sport officials used observation. This result might be beneficial from a practitioner/organizational perspective. That is, unlike the skill acquisition literature, a more general approach to using videos/models could be used across the sport officiating domain thus being more resourceful when conducting clinics and officiating courses.

The research is not without limitations, especially considering this study was exploratory in nature. These limits, however, also become opportunities for future researchers to advance the study of sport officials’ observational learning. A first limitation involves the participants’ sports. While 17 sports were represented in this study, only four sports had at least 25 participants (ice hockey, soccer, lacrosse, and volleyball), with ice hockey having almost double the number of participants compared to the second-most sport, soccer. Thus, the distribution of sports was not equal and should be a priority for future research in this field. In particular, researchers should strive to get representation from a variety of sport types to better understand sport officials’ observation. Second, the survey in this study is not a validated one, though it was deemed appropriate and necessary for this exploratory research in an effort to gain a comprehensive understanding of the ways in which sport officials use observation. Future researchers, though, should consider designing validated instruments that measure and assess sport officials’ observational learning. Such instruments could then be used to significantly improve our understanding of observational learning among sport officials. If sport scientists continue to work in the field of sport officials’ use of observation, and overcome these limitations, it would have tremendous benefits for officials, organizations, and researchers alike.

## Data Availability

The datasets presented in this article are not readily available because participants did not consent to share the data outside the research team. Requests to access the datasets should be directed to dhancock@mun.ca.

## Appendix

I. As an official, I use observation to learn…

1. …proper positioning
2. …about fitness and conditioning
3. …officiating skills and techniques (e.g., signals)
4. …the rules of my sport
5. …how to apply the rules of my sport
6. …to understand officiating strategies
7. …about staying focused during competitions
8. …where to direct my attention
9. …to cope with challenging situations (e.g., high pressure or high anxiety)
10. …an appreciation for how context (e.g., playoffs, rivalries) can influence my decisions
11. …how to adapt my officiating decisions based on the context
12. …to set goals
13. …how to remain confident during performances
14. …pregame routines
15. …to communicate with athletes
16. …to communicate with coaches
17. …to communicate with my officiating partners before a competition
18. …to communicate with my officiating partners during a competition
19. …to communicate with my officiating partners after a competition
20. …how to manage conflicting perspectives with my officiating partners
21. …to improve self-presentation and body language
22. …how to establish presence during a competition
23. …to accept feedback from officials and supervisors

II. As an official, I use observation…

1. …before games I am officiating
2. …during games I am officiating
3. …after games I am officiating
4. …when watching officials on TV
5. …when watching a recording of my competitions
6. …when watching recordings of other officials
7. …when watching other officials in-person
8. …during officiating clinics and meetings
9. …by watching officials who are below my skill level
10. …by watching officials who are at my skill level
11. …by watching officials who are above my skill level
12. …by watching officials correctly execute skills or strategies
13. …by watching officials incorrectly execute skills or strategies
14. …without instruction/cues from other officials or supervisors
15. …with instruction/cues from other officials or supervisors
16. …when I am alone
17. …when I am in an officiating group

III. In an average month as an official, how many…

1. …times do you use observation?
2. …hours do you use observation?
3. …times do you observe yourself?
4. …hours do you observe yourself?
5. …times do you observe other officials in-person?
6. …hours do you observe other officials in-person?
7. …times do you observe other officials on TV?
8. …hours do you observe other officials on TV?
9. …times do you observe recordings of other officials?
10. …hours do you observe recordings of other officials?
